# Bias-corrected serum creatinine from UK Biobank electronic medical records generates an important data resource for kidney function trajectories

**DOI:** 10.1038/s41598-025-85391-7

**Published:** 2025-01-28

**Authors:** Mathias Gorski, Simon Wiegrebe, Ralph Burkhardt, Merle Behr, Helmut Küchenhoff, Klaus J. Stark, Carsten A. Böger, Iris M. Heid

**Affiliations:** 1 Department of Genetic Epidemiology, University of Regensburg, Regensburg, Germany; 2 Statistical Consulting Unit StaBLab, Department of Statistics, Ludwig-Maximilians-Universität, Munich, Germany; 3 Institute of Clinical Chemistry and Laboratory Medicine, University Hospital Regensburg, Regensburg, Germany; 4 Faculty of Informatics and Data Science, University of Regensburg, Regensburg, Germany; 5 Munich Center for Machine Learning (MCML), Munich, Germany; 6 Department of Nephrology, University Hospital Regensburg, Regensburg, Germany; 7 Department of Nephrology, Diabetology and Rheumatology, Kliniken Südostbayern, Traunstein, Germany; 8 KfH Kidney Center Traunstein, Traunstein, Germany

**Keywords:** Nephrology, Kidney, Medical Research, Epidemiology, Kidney, Epidemiology

## Abstract

Loss of kidney function is a substantial personal and public health burden. Kidney function is typically assessed as estimated glomerular filtration rate (eGFR) based on serum creatinine. UK Biobank provides serum creatinine measurements from study center assessments (SC, *n* = 425,147 baseline, *n* = 15,314 with follow-up) and emerging electronic Medical Records (eMR, “GP-clinical”) present a promising resource to augment this data longitudinally. However, it is unclear whether eMR-based and SC-based creatinine values can be used jointly for research on eGFR decline. When comparing eMR-based with SC-based creatinine by calendar year (*n* = 70,231), we found a year-specific multiplicative bias for eMR-based creatinine that decreased over time (factor 0.84 for 2007, 0.97 for 2013). Deriving eGFR based on SC- and bias-corrected eMR-creatinine yielded 454,907 individuals with ≥ 1eGFR assessment (2,102,174 assessments). This included 206,063 individuals with ≥ 2 assessments over up to 60.2 years (median 6.00 assessments, median time = 8.7 years), where we also obtained eMR-based information on kidney disease or renal replacement therapy. We found an annual eGFR decline of 0.11 (95%-CI = 0.10–0.12) versus 1.04 mL/min/1.73m^2^/year (95%-CI = 1.03–1.05) without and with bias-correction, the latter being in line with literature. In summary, our bias-corrected eMR-based creatinine values enabled a 4-fold increased number of eGFR assessments in UK Biobank suitable for kidney function research.

**Supplementary Information:**

The online version contains supplementary material available at 10.1038/s41598-025-85391-7.

## Introduction

Accelerated kidney function decline can lead to renal failure, which necessitates dialysis or kidney transplantation. Age-related decline in kidney function is expected as part of the natural aging process with an average decline of -1mL/min/1.73m^2^ per year of age^1–3^. However, the rate of decline is highly variable in the population and the underlying reasons not fully understood^4,5^. To investigate these reasons, large epidemiological data on kidney function over time in the general population are required. However, such longitudinal datasets on kidney function over time (kidney function trajectories) are sparse. Emerging large biobanks can provide such longitudinal data by integrating information from electronic Medical Records (eMR) on serum creatinine measurements derived from clinical routine.

Data based on eMR are potentially subject to various sources of bias^6^. In the context of eMR-based creatinine values, this might involve selection bias, when individuals with many eMR-based creatinine measurements have the many measurements due to suspected or diagnosed kidney impairment^7,8^. This might also involve technical measurement bias when records of creatinine values extend far into the past and were based on assays before manufacturers have started improving standardization. One initial approach to make old assay creatinine measurements usable for epidemiological studies was to correct values from Jaffe assays used before 2009 by a factor of 0.95^9^. Such a correction can eliminate systematic bias on average, allowing the use of bias-corrected values for epidemiological research.

UK Biobank^10^ provides an opportunity to advance our understanding of kidney function decline in a general adult population. With its large cross-sectional dataset from the study center (SC) assessments, encompassing ~ 500,000 participants, it provides an unprecedented resource for studying cross-sectional kidney function. However, longitudinal UK Biobank data on serum creatinine as kidney function biomarker are currently limited to ~ 15,000 individuals assessed at SC four years after baseline. The recently emerging eMR data from general practitioners (GPs) for UK Biobank participants offer the opportunity to augment existing SC data with longitudinal information. These data were released in September 2019 including multiple serum creatinine measurements over time and recorded kidney-relevant diagnostic codes. The usability of these eMR-based UK Biobank data and its potential for meaningful integration with the SC data is yet to be explored.

Our main objective was thus to augment the existing UK Biobank data on SC-based eGFR with eMR-based data from GPs (“GP-clinical”, application number 20272) to provide a longitudinal data resource for studying kidney function decline. Specifically, we (i) extracted and quality-controlled serum creatinine values derived from UK Biobank GP-eMR data, (ii) compared the eMR-based serum creatinine with SC-based creatinine values; (iii) derived eGFR for the combined eMR- and SC-based creatinine values, (iv) included information on GP-recorded onset of kidney diseases, renal replacement therapy, or other kidney-relevant diagnostic codes, and (v) assessed the utility of these combined data for studying kidney function decline.

## Materials and methods

### UK Biobank data on serum creatinine from SC assessment and GP-clinical

UK Biobank data and SC assessment at baseline and, for a smaller subset of participants, at a 4-year follow-up were described previously^10^. Briefly, UK Biobank is a prospective cohort study that included approximately 500,000 individuals aged 40–69 years at baseline recruited at 22 SCs in the United Kingdom. The SC assessment involved collecting participants’ blood samples and storing aliquots frozen at − 80 °C for further analysis^11^.

Creatinine was measured in serum for all individuals from SC blood drawn at baseline and follow-up in a central laboratory according to standardized protocols (Enzymatic Beckman Coulter AU5800). We obtained these SC-based serum creatinine values (crea_SC_), the date of the SC visit baseline and follow-up (date-of-exam), age at that date (age-at-exam), and sex of participants (data fields 30700, 31, 34, 52 and 53).

Based on the UK Biobank “GP-clinical” table, we obtained raw eMR-based creatinine values (crea_eMR_) via read codes^12^ (Supplementary Table 1). We quality-controlled eMR-based values, excluding technical errors, duplicates, values below the level of detection (LOD) or above the highest recorded value of 6524 µmol/L^13^. Values below the level of quantification (LoQ) were set to the LoQ. Quality-controlled eMR-based values were in µmol/L with recorded measurement dates assumed to be equivalent or close to the date of blood draw (Supplementary Note 1).

The UK Biobank data utilized here included SC data downloaded in September 2019 and information from “GP-clinical” downloaded in May 2023 (i.e. records until September 2017, depending on country Scotland, Wales or England).

### Comparison of crea_SC_ and crea_eMR_ and error model

Next, we merged crea_eMR_ to crea_SC_ for each person by date of blood draw (“date-of-exam”). Crea_eMR_, being derived from clinical routine, were measured by different assays in different laboratories; “GP-clinical” does not contain information on the specific assays. Since in-vitro diagnostic manufacturers were starting to improve standardization of assays around 2009, we investigated the possibility of a systematic technical bias in crea_eMR_ that decreased the closer the measurement date approached 2009 or some years after (assuming a heterogeneous onset of use of standardized products). We considered crea_SC_ gold standard, since these measurements were conducted by one assay and one laboratory via a standardized protocol in the years 2015–2017. We compared distributions, values, and differences of crea_SC_ versus crea_eMR_, focusing on individuals with both from the same calendar year (using crea_eMR_ closest in time to SC blood draw).

We evaluated whether crea_eMR_ exhibited a systematic bias: we assumed normally distributed crea_SC_ and crea_eMR_ on log-scale, $\:X{:=}\text{l}\text{n}\left({crea}_{SC}\:\right)\sim\:N(\mu\:,{\sigma\:}^{2})$ and$\:\:{X}^{*}{:=}\text{l}\text{n}\left({crea}_{eMR}\right)\sim\:N({\mu\:}^{*},\:{\sigma\:}^{*2})$, respectively. We also assumed an additive error consisting of a random component, $\:{E}_{r}^{*}\sim N(0,{\sigma}^{{*}^{2}})$, and a systematic bias, *s*,

$$\:{X}^{*}=X+s+{E}_{r}^{*}.$$

This implies that the expected value of X* is$\:\:E\left[{X}^{*}\right]=E\left[X+s+{E}_{R}^{*}\right]=E\left[X\right]+E\left[s\right]+E\left[{E}_{R}^{*}\right]$, so that$\:\:{\widehat{\mu\:}}^{*}=\:\widehat{\mu\:}+s+0$, yielding$\:\:s={\widehat{\mu\:}}^{*}-\widehat{\mu\:}$. On the original scale, we can derive the geometric means of crea_eMR_ and crea_SC_,$\:\:\text{e}\text{x}\text{p}\left({\mu\:}^{*}\right)$ and$\:\:\text{e}\text{x}\text{p}\left(\mu\:\right)$, respectively. Then the bias is multiplicative and given as the ratio of these geometric means,

$$\:\text{exp}\left(s\right)=\text{e}\text{x}\text{p}\left({\widehat{\mu\:}}^{*}\right)/\text{exp}\left(\mu\:\right).$$

We assumed a differential bias by calendar years (larger in earlier years). Thus, we evaluated differences between crea_eMR_ and crea_SC_ among participants with measurements in the same calendar year. For each calendar year with measurements from both crea_eMR_ and crea_SC_, we estimated $\:{\mu\:}^{*year}$and $\:{\mu\:}^{year}$and quantified the multiplicative bias (on the original scale) per calendar year, $\:\text{exp}\left({s}^{year}\right)=\text{exp}\left({\mu\:}^{*year}\right)/\text{e}\text{x}\text{p}\left({\mu\:}^{year}\right)$

### Bias-corrected crea_eMR_

The above stated error model and bias quantification can also be used to derive bias-corrected crea_eMR_. We used the estimated $\:{s}^{year}$, to correct crea_eMR_ (on ln-scale) measured in the respective calendar year by

$$\:{X}_{corrected}^{*year}={X}^{*}-{s}^{year},$$

where *year* is the calendar year of the crea_eMR_ measurement. The expected value of the corrected crea_eMR_ is then $\:{E[X}_{corrected}^{*year}]=E[{X}^{*}-{s}^{year}]=E[{X}^{*}]-{s}^{year}=(\widehat{\mu\:}+{s}^{year})-{s}^{year}=\widehat{\mu\:}$, thus yielding $\:{X}_{corrected}^{*year}\sim N(\mu\:,{{\sigma}}^{{*}^{2}})$, with a purely random error compared to crea_SC_, $\:{X}_{corrected}^{*year}=X+{E}_{r}^{*}$.

For calendar years without individuals that had both crea_eMR_ and crea_SC_ available to estimate the year-specific correction factor, we used$\:{\:s}^{year}=0.5*{s}^{year+1}+{s}^{year-1}$, or $\:{s}^{year}\:$from the first or last year. We utilized bias-corrected crea_eMR_ for further analyses and merged them to crea_SC_ by person and date-of-exam.

### Generating the eGFR data based on crea_SC_ and crea_eMR_

We utilized the combined data of crea_SC_ and bias-corrected crea_eMR_ to derive eGFR via the most recent, race-term free, formula^14^. For this, we used age-at-exam (i.e. difference between date-at-exam and date-of-birth) and sex (reported at baseline SC visit). We defined Chronic Kidney Disease (CKD) as eGFR < 60 mL/min/1.73m^2^).

### Integrating GP-eMR data on kidney function relevant diagnostic codes

We also extracted recorded information on kidney function relevant diagnostic codes from UK Biobank “GP-clinical” and the corresponding date: (i) severe kidney disease (End-stage Kidney Disease, ESKD; Acute Kidney Injury, AKI), (ii) intervention (nephrectomy, dialysis, kidney transplantation) and (iii) other conditions relevant to kidney function (pregnancy, diabetes). For this, we used the Coding System Lookups and Mappings Dictionary (Version 3, May 2021) to identify the respective Read v2 and CTV3 read codes (Supplementary Table 1, Supplementary Note 2). These data were then merged with serum creatinine data based on date-of-exam.

### Statistical analyses on final dataset

For our final dataset including kidney-relevant diagnostic codes and eGFR based on crea_SC_ or bias-corrected crea_eMR_, we derived descriptive statistics of the included UK Biobank study participants. For this, we used SC-baseline information regarding lifestyle (smoking; body-mass-index, BMI), diabetes (HbA1c > 6.5%, self-report or medication) and CKD status (SC-based eGFR).

We evaluated a potential selection of UK Biobank participants into the “GP-clinical”. We thus compared descriptive statistics of individuals in “GP-clinical” (GP-clinical members) versus individuals not in “GP-clinical” regarding lifestyle, diabetes or CKD status assessed at SC-baseline. We also compared descriptive statistics between individuals with many versus few assessments regarding (≥ 10 versus ≥ 2 eGFR assessments).

We then investigated whether the derived data were suitable for research on eGFR decline. For this, we restricted to individuals with ≥ 2 eGFR assessments at least one year apart, censoring eGFR values after onset of kidney-relevant events (for pregnancy, excluding values ± 6 months before and after). We derived person-specific annual eGFR decline as difference between last and 1st eGFR assessment divided by the number of years-in-between. First, we derived mean annual eGFR decline (and 95%-CI) with and without bias-correction for crea_eMR_. Second, we used multivariable linear regression to quantify risk factor association with annual eGFR decline. For this, we applied two models: (i) one model including age at first assessment (centered at 40 years), sex, and one genetic variant known for strong association with eGFR decline^15^ (UMOD, rs77924615) and (ii) another model without the genetic variant but adding smoking (self-report), obesity (body-mass-index, BMI, ≥ versus < 30 kg/m^2^; measured weight and height) and diabetes (self-report; all from SC-baseline, assuming that this is a reasonable proxy for these risk factors at first assessment). Third, we restricted to individuals with any AKI record and estimated mean annual eGFR decline (and 95%-CI) around an incident AKI event using eGFR assessments ≥ 6 months (as close as possible to the 6 months) before and after AKI.

## Results

### Serum creatinine measurements from the SC visits and GP-eMR

When extracting crea_SC_ from the baseline SC visit (year of exam 2006–2010), we yielded measurements for 425,147 individuals. For 16,446 individuals, crea_SC_ was available from the follow-up SC visit (year of exam 2012–2013). Together, this resulted in 15,314 individuals with 2 creatinine values over up to 6.1 years (median time = 4.4 years) and 410,965 individuals with exactly one creatinine value.

When extracting crea_eMR_ from “GP-clinical”^12^, we yielded 1,701,710 raw creatinine values for 199,482 individuals. After quality control, this resulted in 1,660,581 crea_eMR_ values for 199,396 individuals (Supplementary Fig. 1). This included 23,188 individuals with exactly 2 creatinine measurements and 151,728 with ≥ 3 measurements (median number of measurements per person = 7.00, max = 288). Year of exam (i.e. year of measurement) was as early as 1950 up to 2017 resulting in a time between 1st and last measurement of up to 60.2 years (median time = 8.12 years).

**Fig. 1:**
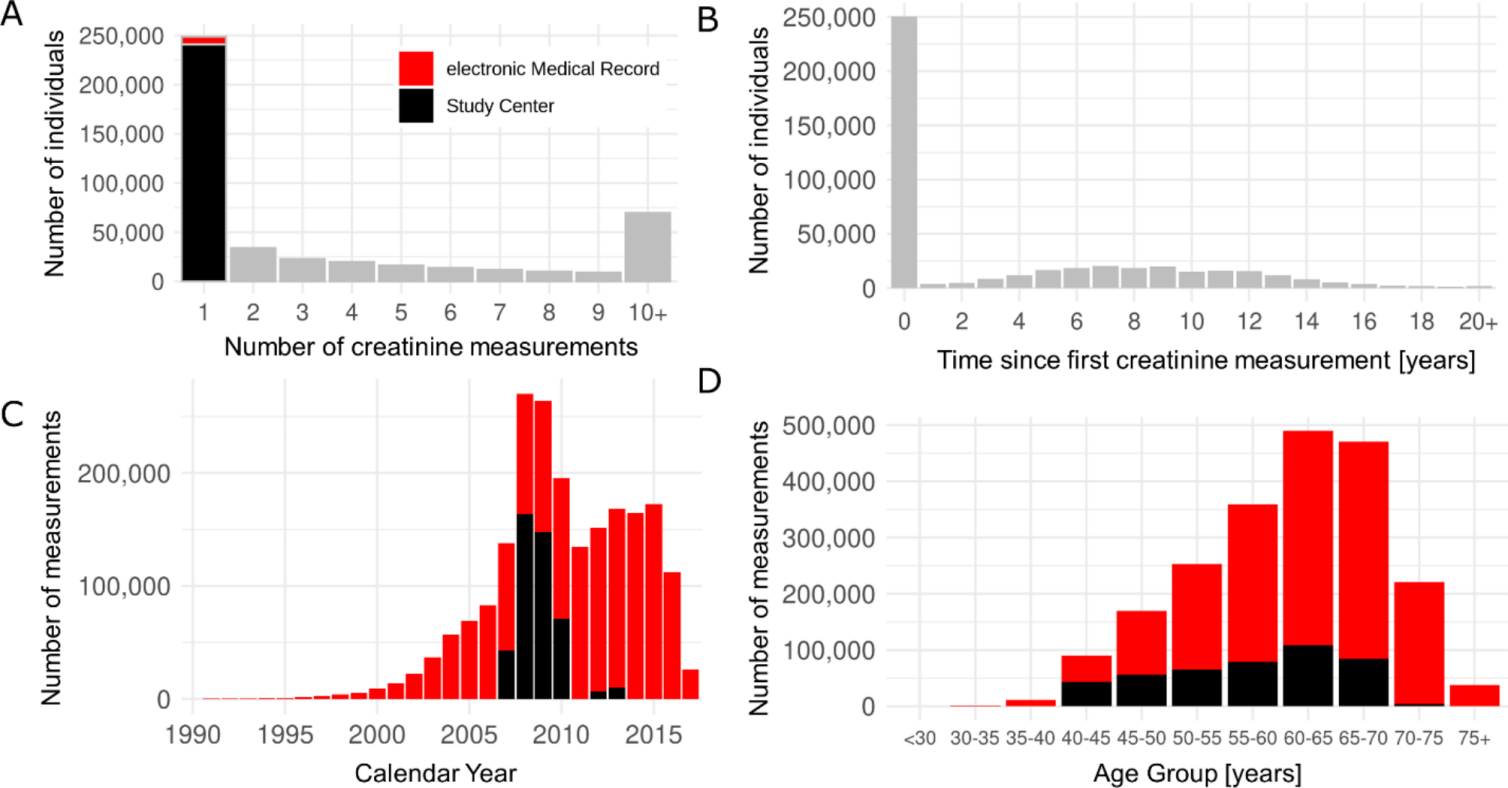
Number of creatinine measurements from study center (SC) and electronic Medical Records (eMR). When combining SC- and eMR-based creatinine values after quality-control, we obtained 2,102,174 creatinine values from 454,907 individuals. We show (**A**) the distribution of individuals by their number of available creatinine measurements over time (*n* = 248,844 with = 1 measurement indicated as SC- or eMR-derived in black or red, respectively; *n* = 206,063 with ≥ 2 measurements over time in gray), (**B**) the distribution of individuals by the time between 1st and last measurement (“assessment period”), (**C**) the distribution of the number of measurements by calendar year of assessment, and (**D**) the distribution of the number of measurements available by age groups (age-at-exam, “exam” referring to the GP-record for eMR or to the examination at the SC visit). For **C**&**D**, red colour represents eMR and black colour represents SC data.

When merging crea_eMR_ and crea_SC_ by date-of-exam (i.e. date of measurement for eMR; date of SC-visit for SC), we yielded 2,102,174 creatinine values for 454,907 individuals. Thus, crea_eMR_ substantially extended the longitudinal information on eGFR for UK Biobank participants from 4 years to up to 60.2 years and from a maximum of 2 to up to 289 measurements per person (Fig. 1A, B). The dataset included 206,063 individuals with ≥ 2 measurements (median time between 1st and last assessment = 8.71 years; median number of measurements per person = 6.00, Fig. 1C, D).

### Bias-corrected crea_eMR_ values

**Fig. 2:**
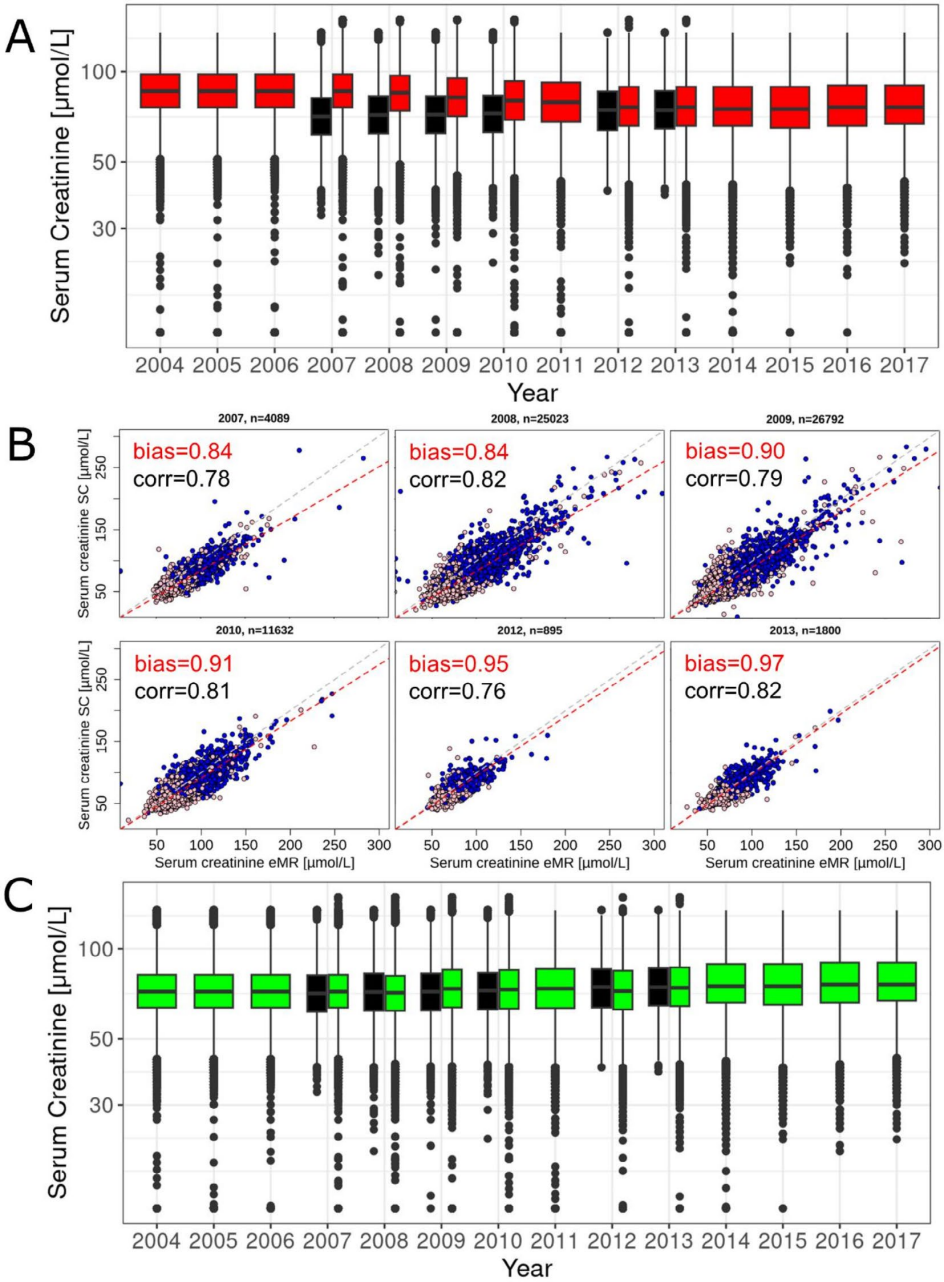
Bias-correction of serum creatinine values from electronic Medical Records (eMR) data. We contrasted serum creatinine values from study center (SC) versus eMR by calendar year-of-exam (i.e. year of blood draw & measurement for eMR, year of blood draw for SC with centralized measurement in 2007–2010 and 2012–2013). (**A**) We show the distributions of creatinine from eMR (red; quality-controlled, not bias-corrected) and SC (black). (**B**) We show creatinine values from SC- versus eMR (quality-controlled, not bias-corrected) among 70,231 individuals with both measurements from the same calendar year (using the eMR-creatinine value closest in time to the SC-value). Also shown is the estimated bias (‘bias’, i.e. ratio of geometric means of SC-values and eMR-values) and Spearman correlation coefficient (‘corr’). Grey lines indicate the identity. Red lines indicate the line through null with a bias slope. Sex is color-coded (blue: men, pink: women). (**C**) Shown are the distributions of bias-corrected creatinine from eMR (green; bias-corrected) and SC (black). The year-specific bias estimate was used as correction factor; for other years, we used proxies (2011: 0.93 derived as exp of average between ln(bias) for 2010 and 2012; years before 2007: 0.84; years after 2013: 1.0 - no correction).

We hypothesized that crea_eMR_ measurements conducted in years substantially earlier than 2009 were subject to a measurement bias. We further hypothesized that this bias became smaller the closer the measurement year approached 2009, where in-vitro diagnostic manufacturers started to improve standardization of creatinine assays. To investigate this, we focused on the 70,231 individuals with both crea_SC_ and crea_eMR_ available from the same calendar year (i.e. year-of-exam 2007–2010 and 2011&2012). We observed higher median crea_eMR_ compared to crea_SC_ in earlier years, which gradually converged towards nearly identical median values in 2013 (Fig. 2A, Supplementary Fig. 2).

We quantified the bias of crea_eMR_ based on an additive error model on the log-scale as the difference between mean ln(crea_eMR_) and mean ln(crea_SC_) per calendar year, $\:{s}^{year}$. This can also be interpreted as multiplicative bias on the original scale, $\:\text{e}\text{x}\text{p}\left({s}^{year}\right)$, derived as ratio of the geometric means of crea_eMR_ and crea_SC_ (“Methods”). We found a bias of factor 0.84 for the year 2007, which converged to near unity until year 2013 (0.84, 0.84, 0.90, 0.91, 0.95 and 0.97 for the years 2007–2010 and 2012–2013, respectively; Fig. 2B). We obtained nearly the same bias estimates when winsorizing extreme values (beyond mean ± 3 standard deviations on log-scale:$\:\:\text{e}\text{x}\text{p}\left({s}^{year}\right)$ =0.84, 0.84, 0.90, 0.91, 0.95 and 0.98), documenting the lack of influence of extreme values.

In order to obtain bias-corrected crea_eMR_ for all individuals, we used the derived year-specific correction factors $\:\text{e}\text{x}\text{p}\left({s}^{year}\right)$ to correct crea_eMR_ measurements from the respective year (i.e. 2007–2010, 2012 and 2013). For years where the correction factor could not be estimated directly, we used proxy correction factors: (i) average of 2010 and 2012 on log-scale for 2011 (0.93); (ii) the 2007 factor for measurements before 2007 (0.84), (iii) and 1.0 (no correction) for measurements after 2013. When comparing the year-specific distributions of corrected crea_eMR_ with crea_SC_ distributions, we now found similar distributions and median values (Fig. 2C).

### Description of the resulting UK Biobank dataset on 454,907 individuals with eGFR including eGFR trajectories

**Fig. 3:**
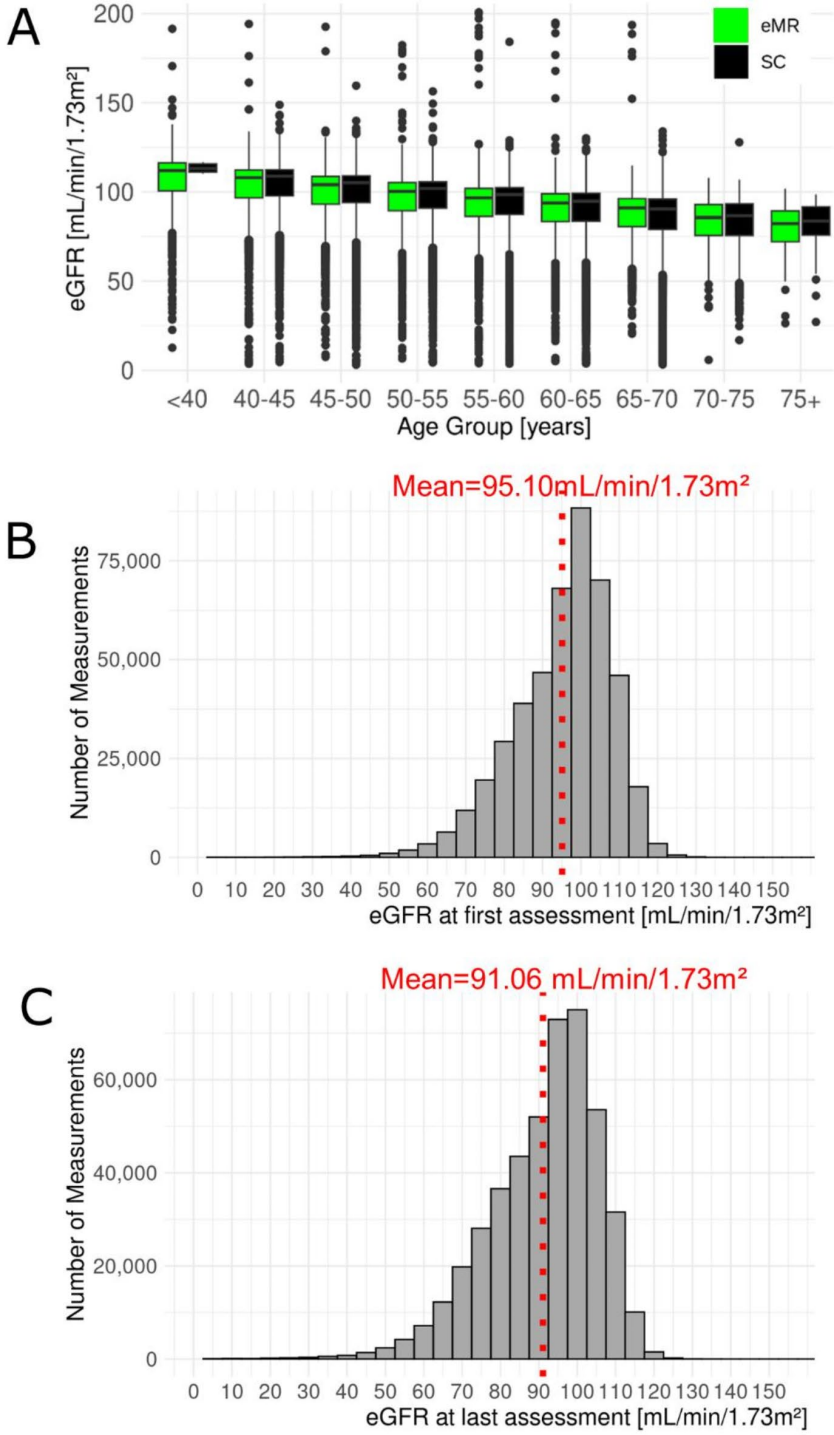
Descriptive of combined data from electronic Medical Records (eMR)- and study center (SC)-based eGFR. The dataset for eGFR from SC and eMR combined comprised 454,907 individuals with ≥ 1 eGFR assessment and overall 2,102,174 assessments of eGFR derived from bias-corrected creatinine. (**A**) Shown are age-group-specific eGFR distributions^14^ based on data from SC (black) and eMR (bias-corrected, green). This was limited to the 1st eGFR assessment from SC or eMR, respectively. We also show eGFR distributions at **(B)** first and **(C)** last assessment (omitting 48 extreme values ≥ 150mL/min/1.73m^2^).

Next, we merged crea_SC_ and crea_eMR_ by date-of-exam and derived eGFR using CKD-EPI 2021 ^14^. When comparing crea_eMR_-based eGFR (eGFR_eMR_) with crea_SC_-based eGFR (eGFR_SC_), we observed a bias in eGFR_eMR_ without bias-correction of crea_eMR_ and no bias with correction (Supplementary Figs. 3, 4). We used bias-corrected crea_eMR_ and corresponding eGFR_eMR_ for the following. We found that the distributions of eGFR_eMR_ and eGFR_SC_ were comparable by 5-year age groups (Fig. 3A).

The final data comprised 454,907 individuals with ≥ 1 eGFR assessment based on crea_SC_ and/or crea_eMR_ (54.2% women) and with overall 2,102,174 eGFR assessments (Table 1). Mean age was 55.9 years and 59.8 years at 1st and last eGFR assessment respectively; mean eGFR was 95.1 and 91.1 mL/min/1.73m^2^, respectively (Fig. 3B, C). The data consisted of (i) 248,844 individuals with = 1 eGFR assessment, mostly from SC baseline visit (98.26% from SC baseline, 0.32% from SC follow-up, 2.21% from eMR), (ii) 33,851 individuals with = 2 assessments (60.0% of these with one assessment from SC and one from eMR), and (iii) 172,212 with ≥ 3 assessments (i.e. ≥k-2 assessments from eMR, k being the number of assessments per person). Thus, the dataset now included 33,851 + 172,212 = 206,063 individuals with repeated eGFR values over time (eGFR trajectories).

**Table 1:** Participant descriptive statistics for the UK Biobank data on eGFR including eGFR trajectories.We show participant characteristics using information from the study center (SC) visit at baseline and descriptive statistics of temporal aspects of the combined SC- and (bias-corrected) eMR-based data on eGFR^14^. These descriptive statistics are shown for all individuals and restricted to individuals with ≥2 or ≥10 eGFR assessments (further details in Supplementary Table 2). In the table header, ‘n’ denotes the number of individuals and ‘m’ denotes the number of eGFR assessments. Presented are mean and standard deviations, if not stated otherwise.

	All Data (*n* = 454,907, m = 2,102,174)	≥ 2 eGFR (*n* = 206,063, m = 1,853,330)	≥ 10 eGFR (*n* = 69,506, m = 1,234,508)
Characteristics from SC-baseline*			
Age - years	57.3 ± 8.1	57.9 ± 7.9	60.7 ± 6.9
Sex – female (%)	246,480 (54.2)	111,528 (54.1)	34,389 (49.5)
Ancestry - European (%)	414,097 (91.0)	180,359 (87.5)	59,909 (86.2)
Smoking status ** (%)	204,051 (44.9)	93,244 (45.3)	34,771 (50.1)
BMI **– kg/m^2^	27.4 ± 4.8	27.6 ± 4.9	29.1 ± 5.3
Diabetes – yes (%)	19,479 (4.5)	9,579 (4.9)	8,219 (12.6)
eGFR - mL/min/1.73m^2^	94.1 ± 13.2	93.6 ± 13.2	90.5 ± 14.5
Chronic Kidney Disease*** – yes (%)	7,258 (1.6)	3,437 (1.8)	2,284 (3.6)
Combined SC- and eMR-based assessments			
Age-at-first-exam^‡^ - years	55.9 ± 8.2	54.7 ± 8.0	55.7 ± 7.3
Age-at-last-exam^‡^ - years	59.8 ± 8.9	63.44 ± 8.5	67.6 ± 6.9
time between 1st and last eGFR assessment - median (max) - years	0.0 (60.2)	8.5 (60.2)	11.7 (60.2)
Calendar year of exam^‡^ - min to max	1950 to 2017	1950–2017	1950–2017
#eGFR assessments per person – median [IQR]	1.0 [1.0–5.0]	6.0 [3.0–12.0]	15.0 [12.0–20.0]
eGFR-first-exam - mL/min/1.73m^2^	95.1 ± 12.9	95.9 ± 12.6	94.2 ± 13.3
eGFR-last-exam - mL/min/1.73m^2^	91.1 ± 14.4	86.9 ± 14.8	81.5 ± 16.5
Chronic Kidney Disease*** – yes (%)	23,035 (5.1)	19,191 (9.3)	14,212 (20.5)

BMI = Body Mass Index, eGFR = estimated Glomerular Filtration Rate.

*Obtained from SC-baseline (data fields 31, 34, 52, 53, 2443, 20003, 20116, 21001, 30700, and 30750).

**Among individuals where this variable is available (*n* = 454,361 for smoking status 0 = never smoking, 1 = current or previous smoking, *n* = 452,721 for BMI).

*** Individuals having eGFR < 60 mL/min/1.73m^2^ for at least one timepoint.

^‡^ “Exam” is the examination in SC or the examination by the GP, assuming that the date of the eMR-record is the same date as the GP-exam.

The characteristics of these individuals with ≥ 2 eGFR assessments (regarding age, %women, %smoking, BMI, eGFR, and %CKD from SC-baseline; *n* = 206,063) were similar as in the overall data, but individuals with ≥ 10 eGFR assessments (*n* = 69,506) were older, with higher BMI, lower eGFR, higher %CKD (Table 1).

This difference between individuals with many versus few assessments was also observable when restricting to individuals that were part of “GP-clinical” (GP-clinical members, i.e. individuals with ≥ 1 eGFR assessed from crea_eMR_; *n* = 199,396), while there was no difference between GP-clinical members and individuals that were not in GP-clinical (*n* = 199,396 versus 255,511, respectively; Supplementary Table 2). Thus, we found evidence for a selection towards older and less healthy individuals among those with many crea_EMR_ values over time compared to fewer crea_eMR_ values, in line with literature^7,8^. However, there was no selection observed for being a GP-clinical member versus all UK Biobank participants.

### Description of UK Biobank eGFR trajectories data for 206,063 individuals augmented with kidney function relevant diagnostic codes

The generated UK Biobank eGFR trajectories dataset consisted of 206,063 individuals with ≥ 2eGFR assessments over time (total of 1,853,330 eGFR assessments; Table 1). Median time between 1st and last assessment was 8.5 years (maximum 60.2 years), median number of eGFR assessments over time per person was 6.0 (maximum 289 assessments). Most of these individuals had ≥ 1 eGFR assessed from crea_eMR_, (*n* = 195,885; 95.1%), thus were members of GP-clinical.

For GP-clinical members, we extracted records of kidney disease or intervention (AKI, ESKD, Nephrectomy, Dialysis, kidney transplantation) as well as other kidney function related codes (pregnancy, Diabetes) and the corresponding date from UK Biobank “GP-clinical” (Supplementary Table 1). We merged this information to the eGFR trajectories by date-of-exam. For the 195,885 GP-clinical members with ≥ 2 eGFR assessments over time, we yielded 74,501 records of any kidney disease, intervention, pregnancy or diabetes. These included 94, 49, 33, 30, 428 individuals with any record of AKI, ESKD, kidney transplant, dialysis, or nephrectomy before the 1st eGFR assessment and 562, 228, 180, 142, or 536 after (Table 2).

**Table 2:** Recorded kidney function relevant diagnostic codes among UK Biobank individuals in GP-clinical. We show descriptive statistics on recorded kidney disease, renal replacement therapy, or other diagnostic codes that are kidney function relevant. We show this for all individuals in “GP-clinical” (i.e. have ≥1 eGFR assessment from crea_eMR_; n=199,396) and restricted to individuals with ≥2 or ≥10 eGFR assessments. In the table header, ‘n’ denotes the number of individuals. We differentiated between diagnostic codes recorded before and after the first available creatinine measurement. If a diagnostic code is reported multiple times for an individual, it is counted once in each category. Results are presented as absolute count and as percentage in parentheses.

Data source	≥ 1 eGFR assessment and in GP-clinical (*n* = 199,396)	≥ 2 eGFR assessments and in GP-clinical (*n* = 195,885)	≥ 10 eGFR assessments and in GP-clinical (*n* = 69,506)
Diagnostic codes reported before 1st eGFR assessment			
Acute Kidney Injury (%)	94 (< 0.1%)	94 (< 0.1%)	51 (< 0.1%)
End-Stage-Kidney-Disease (%)	49 (< 0.1%)	49 (< 0.1%)	35 (< 0.1%)
Kidney transplant (%)	33 (< 0.1%)	33 (< 0.1%)	24 (< 0.1%)
Dialysis (%)	30 (< 0.1%)	30 (< 0.1%)	22 (< 0.1%)
Nephrectomy (%)	430 (0.2%)	428 (0.2%)	236 (0.3%)
Pregnancy (%)	6,003 (3.0%)	5,848 (3.0%)	1,208 (1.7%)
Diabetes (%)	15,957 (8.0%)	15,758 (8.0%)	7,329 (10.5%)
Diagnostic codes reported after 1st eGFR assessment			
Acute Kidney Injury (%)	563 (0.3%)	562 (0.3%)	461 (0.7%)
End-Stage-Kidney-Disease (%)	228 (0.1%)	228 (0.1%)	194 (0.3%)
Kidney transplant (%)	180 (< 0.1%)	180 (< 0.1%)	140 (0.2%)
Dialysis (%)	142 (< 0.1%)	142 (< 0.1%)	106 (0.2%)
Nephrectomy (%)	540 (0.3%)	536 (0.3%)	352 (0.5%)
Pregnancy (%)	656 (0.3%)	634 (0.3%)	148 (0.2%)
Diabetes (%)	15,063 (7.6%)	14,903 (7.6%)	7,482 (10.8%)

The proportion of individuals with kidney disease or intervention recorded after the 1st eGFR assessment was slightly lower among individuals with ≥ 2 eGFR assessments than when restricting to individuals with ≥ 10 assessments (Table 2; e.g. 0.3% versus 0.7% for AKI). This data can be utilized e.g. for focused analyses on patients using trajectories after AKI, or for analyses on individuals without AKI, ESKD, or intervention affecting eGFR.

### Some aspects of UK Biobank eGFR trajectories exemplifying its utility

To document the utility of the generated longitudinal eGFR data with bias-corrected crea_eMR_, we estimated annual eGFR decline without and with bias-correction. For this, we restricted to the 202,794 individuals with ≥ 2 eGFR assessments at least 1 year in-between, censoring individuals after observed eGFR < 15 mL/min/1.73m^2^ or recorded kidney disease or renal replacement therapy (or nephrectomy of any causes). In the data integrating eMR- and SC-based eGFR, we estimated mean annual decline (difference between last and 1st eGFR assessment divided by years in-between) at 1.04 mL/min/1.73m^2^ per year (95%-CI = 1.03–1.05) with bias correction and 0.11 mL/min/1.73m^2^ per year (95%-CI = 0.10–0.12) without. This documented a plausible eGFR decline in line with literature^1–3^ in the data with bias correction, but not without.

We also evaluated the impact of bias-correction on risk factor association with annual decline using multivariable linear regression (“Methods”, considering a risk factor associated when the 95% CI did not overlap zero): (i) in a model with age at first assessment, sex, and the genetic variant, rs77924615, known for its large effect on eGFR^16^ and eGFR decline^15^, we observed a slightly deflated genetic effect size without versus with bias-correction (Table 3); (ii) in a model without the genetic variant but adding smoking, obesity, and diabetes, we found smoking and diabetes (but not obesity independent of diabetes) associated with increased annual decline in the data with bias-correction, but smoking was not associated in the data without bias-correction. Thus, the estimation of risk factor associations appeared to be affected by the bias, but was less affected than the estimation of mean eGFR decline.

**Table 3:** Impact of bias-correction on risk factor association with annual decline. We were interested in the influence of the bias-correction of creatinine from electronic medical records (eMR) on risk factor association with annual decline. For this, we present the results of a multivariable linear regression model examining annual decline computed as annualized difference between two eGFR assessments as the outcome. The model includes the covariables (i) age at first exam, sex, and the genetic variant rs77924615^15,16^ and (ii) adding smoking, obesity, and diabetes at SC baseline (without the genetic variant). We present beta-estimates [95%-CIs] of (left side) not-bias corrected creatinine and (right side) bias-corrected creatinine.

	Not bias-corrected creatinine	Bias-corrected creatinine
	Beta [95%-CI]	Beta [95%-CI]
Model: annual decline ~ Age + Sex + rs77924615 (*n* = 187,901; m = 1,694,341)		
(Intercept)	-0.203 (-0.229, -0.176)	0.947 (0.926, 0.968)
Age*	0.019 (0.017, 0.020)	0.005 (0.003, 0.006)
Sex**	0.140 (0.118, 0.162)	0.108 (0.091, 0.126)
rs77924615 (G)***	0.034 (0.014, 0.054)	0.052 (0.036, 0.068)
Model: annual decline ~ Age + Sex + Smoking + Obesity + Diabetes (*n* = 191,370; m = 1,727,875)		
(Intercept)	-0.203 (-0.230, -0.176)	0.905 (0.883, 0.926)
Age*	0.019 (0.017, 0.020)	0.004 (0.003, 0.005)
Sex**	0.131 (0.109, 0.154)	0.092 (0.074, 0.110)
Smoking^‡^	-0.013 (-0.036, 0.009)	0.026 (0.008, 0.044)
Obesity^†^	-0.066 (-0.091, -0.040)	0.010 (-0.010, 0.030)
Diabetes^¥^	0.344 (0.292, 0.396)	0.383 (0.342, 0.424)

Beta [95% CI] = Effect [Lower, upper 95%-confidence interval: Beta ± 1.96*SE].

*Age = age at first assessment centered at 40 years (age – 40 years).

**Sex = 0:women, 1:men.

***rs77924615 is a single nucleotide polymorphism near the *UMOD* gene, imputed with UK10K plus 1000 Genomes Phase 3 reference haplotypes. Imputed allele dosages were used for the association analysis. The coded allele is the reported annual decline increasing allele G.

^‡^Smoking: 0 = never smoking, 1 = current or previous smoking.

^†^Obesity: 0 = BMI < 30 kg/m^2^, 1 = BMI ≥ 30 kg/m^2^.

^¥^Diabetes: 0 = no diabetes, 1 = diabetes.

**Fig. 4:**
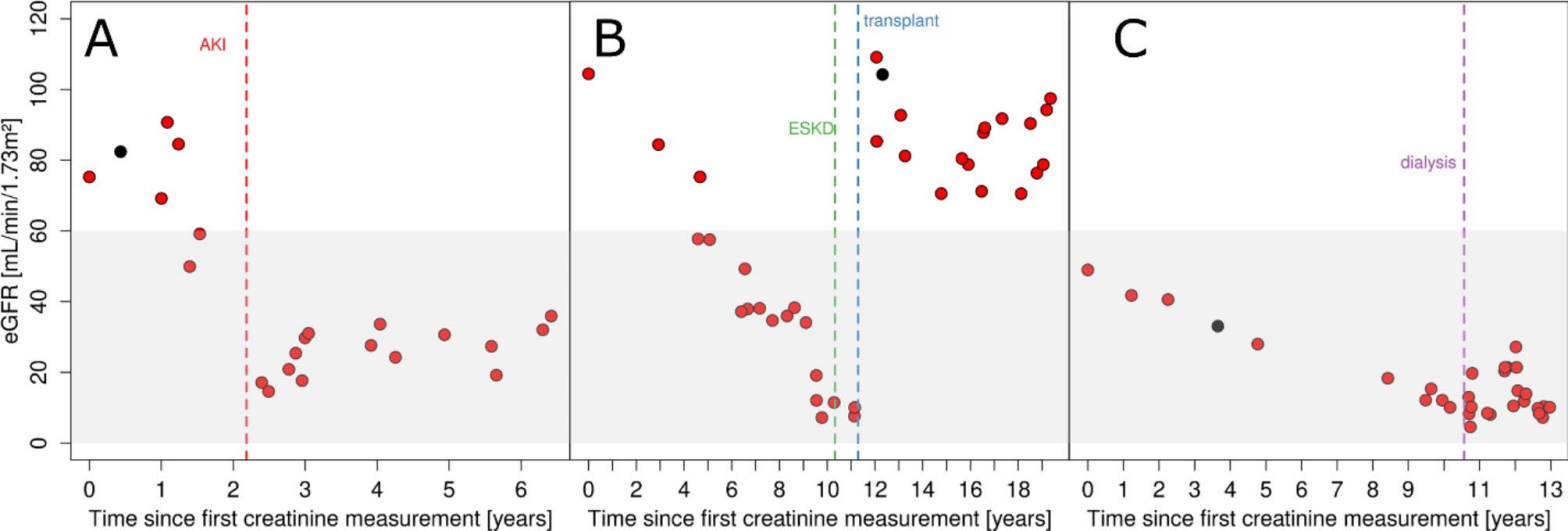
Showcases of eGFR trajectories for individuals with recorded kidney-relevant kidney data. Shown are three examples of eGFR trajectories: (**A**) a trajectory with an episode of Acute Kidney Injury (AKI) and subsequent partial eGFR recovery, (**B**) a trajectory with eGFR decline down to 10 mL/min/1.73m^2^, a recording of ESKD, and eGFR recovery after kidney transplantation and (**C**) a trajectory with eGFR decline down to 15 mL/min/1.73m^2^ and a record of dialysis. The x-axis depicts the time since 1st eGFR assessment in years, the y-axis depicts the eGFR value in mL/min/1.73m^2^. Red and black dots indicated whether the eGFR was based on electronic Medical Records or from study center assessment, respectively. The shaded area indicates eGFR < 60 mL/min/1.73m^2^ (i.e. defining Chronic Kidney Disease, CKD). Events of ESKD, AKI and transplant are visualized at vertical dotted lines in green, red and blue, respectively.

To provide a further example, we evaluated whether the (bias-corrected) eGFR trajectories reflected impaired kidney function during AKI: for the 354 individuals with incident AKI and ≥ 1 eGFR assessment 6 months before and after AKI, mean annual eGFR decline was four times larger than the overall decline (3.93 mL/min/1.73m^2^ per year, 95%-CI = 2.82–5.05). An example of an eGFR trajectory with such a rapid eGFR decline around AKI is illustrated in Fig. 4A. Two further examples illustrate eGFR trajectories with recorded ESKD (also eGFR < 15 mL/min/1.73m^2^) followed by transplantation and of an individual with dialysis, respectively (Fig. 4B, C).

Overall, we curated UK Biobank data and generated new variables for 454,907 individuals with at least one eGFR assessment, including 206,063 individuals with eGFR trajectories (i.e. at least 2 eGFR assessments) and kidney-relevant diagnostic codes.

## Discussion

**Fig. 5:**
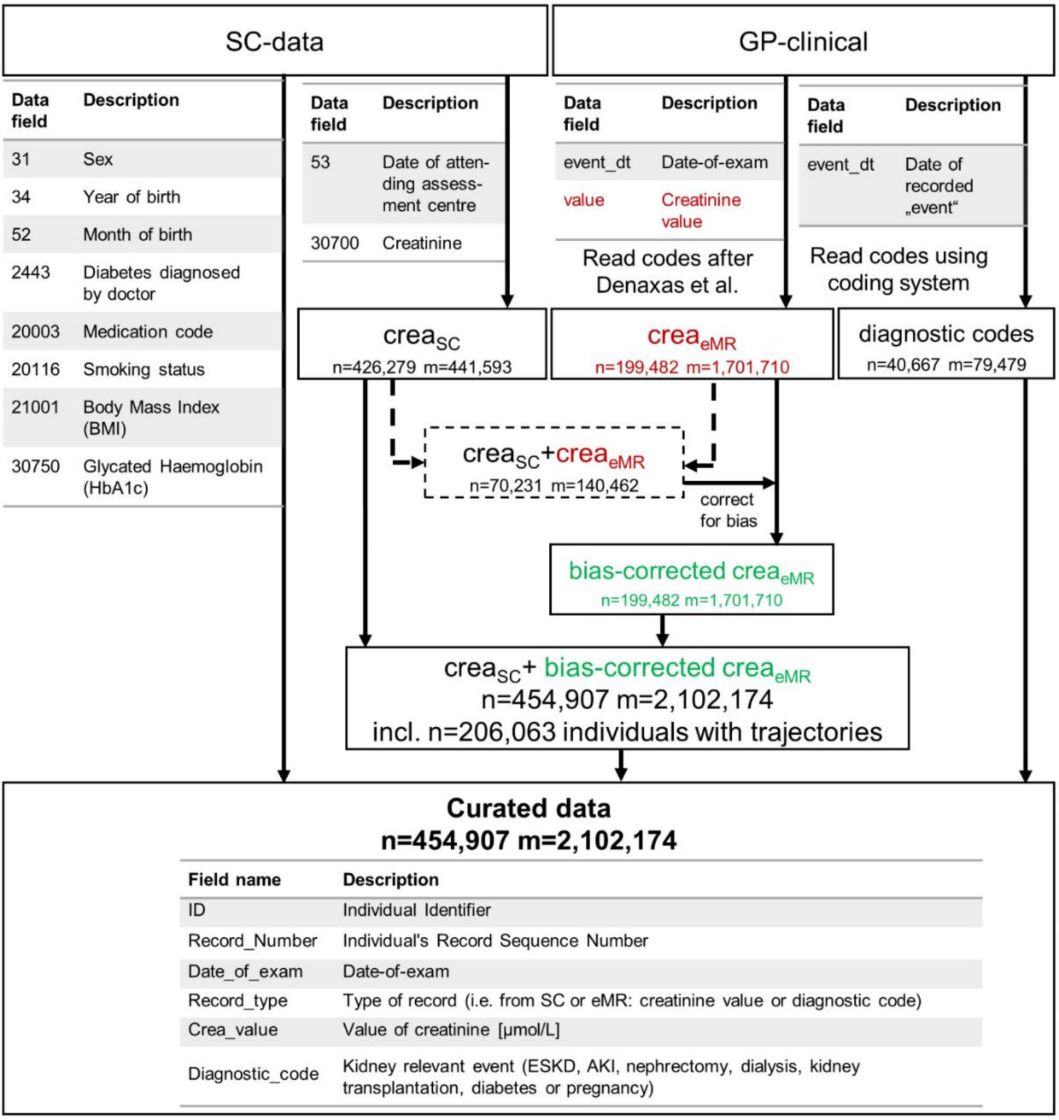
Workflow from raw data to the curated data. This schematic illustrates the analytical flow of the data generation. We show which variables were used from study center (SC) regarding creatinine and other SC-based values and what eMR data were used from GP-clinical. We used eMR-data for creatinine^12^ and other kidney function relevant diagnostic codes (onset of Acute kidney injury, AKI or Endstage kidney disease, ESKD; interventions nephrectomy, dialysis or transplantation and other kidney relevant codes on Diabetes^17^ or pregancy). Date of recorded “event” was used from data field “event_dt’. We derived calendar-specific bias correction factor for crea_eMR_ in individuals with crea_eMR_ and crea_SC_ available in the same calendar year (dashed lines) and used proxy correction factors for other calendar years. We combined crea_SC_ and bias-corrected crea_eMR_ with other SC-based data and eMR-based diagnostic codes. ‘n’ denotes the number of individuals and ‘m’ the number creatinine measures. We colour-coded uncorrected eMR-creatinine (red) and bias-corrected eMR-creatinine (green).

With this work, we present quality-controlled UK Biobank data of 2,102,174 SC- and eMR-based creatinine measurements and calculated eGFR in 454,907 individuals. For 206,063 of these individuals, eGFR trajectories are available with two or more eGFR assessments over time with a medium time between 1st and last assessment of 8.5 years. Purely SC-based information on creatinine and eGFR at two time points was available for 15,314 individuals with medium follow-up time of 4.4 years. Thus, by including eMR-based information on serum creatinine from GP-clinical, we extended the longitudinal UK Biobank data on eGFR by > 10-fold in terms of number of individuals and > 2-fold in terms of medium length of follow-up. We augmented this dataset with kidney-relevant diagnostic codes from GP-clinical (Fig. 5).

One key aspect of integrating eMR-based creatinine measurements to SC-based measurements in UK Biobank was the quantification and accounting for systematic measurement error. The crea_eMR_ values stemmed from routine outpatient care and were prone to bias inherent to using creatinine assays before improved standardization. UK Biobank provided the opportunity to compare these measurements with crea_SC_, which were measured centrally in 2019 using standardized biobanked serum and a modern enzymatic assay calibrated to National Institute Standardized Technology (NIST, reference material 967). The observed bias of factor 0.84 for calendar years 2007 and 2008, which diminished to factor 0.90 for the year 2009 was in line with correcting values from Jaffe assays used before 2009^9^. The observed bias of 0.95 and 0.97 for the years 2012 and 2013 reflected the increased use of standardized assay. When using these bias estimates to correct crea_eMR_ values, we were able to show that the average crea_eMR_ and derived eGFR_eMR_ aligned with the average crea_SC_ and eGFR_SC_, respectively. Furthermore, we showed that the estimated annual eGFR decline was 0.11 versus 1.04 mL/min/1.73m^2^ per year without and with the bias-correction, with the latter being in line with literature^1–3^. When exploring the impact of bias-correction on risk factor association with eGFR decline, we found slightly deflated risk factor estimates in the data without compared to with bias-correction, but less impact than for the estimation of mean eGFR decline. This underscored the utility of this bias-correction when integrating eMR-based eGFR from past creatinine measurements in UK Biobank GP-clinical. The notable change before and after correcting for bias also calls for cautious interpretation of eGFR decline estimates in other data when creatinine values from standardized assessments via SC are integrated with values from eMR obtained before laboratory standards were implemented and assays were unknown. Overall, we provide an approach to correct for systematic measurement error in eMR-based serum creatinine, when gold standard measurements of creatinine were available. We considered SC-based creatinine measurements gold standard, since these measurements were all conducted with one assay in one laboratory in a standardized way.

For studies with past eMR-based creatinine measurements but no gold standard measurements, this might not be fully generalizable. When the studies’ eMR-based creatinine measurements are derived in a comparable fashion as in UK Biobank ‘GP-clinical’, a similar calendar year dependent bias for measurements before 2009 might apply. However, the generalizability of the derived correction factors to other countries and health care systems is probably limited. Still, the here presented correction factors might provide a reasonable proxy of this bias, at least they give a notion of the extent of the problem, and accounting for some bias might be better than assuming no bias. Further evaluations of the bias in creatinine measurements before laboratory standards became available in other GP-eMR data in UK or other countries would be warranted. This extends to other biomarkers of kidney function like Cystatin C – or, actually, all laboratory biomarkers.

There are some limitations to be acknowledged. Our bias-correction works on average, which makes the data usable to estimate average kidney function decline or risk factor association. However, the bias-correction does not yield valid person-specific corrected creatinine and eGFR values, since the specific assay for an individual’s measurements was unknown. Furthermore, the bias quantification was only possible in calendar years 2007 to 2010 and from 2012 to 2013 with both crea_eMR_ and crea_SC_ values available and the correction factors had to be extrapolated for any other years. This does not reflect the large timespan of up to 60 years and measurements made as early as 1950, which implies changes in technology and laboratory practice that are not covered by our evaluations. Finally, we have only applied one approach to bias-quantification. For example, we derived an error model and bias per calendar year, while other error models might use the time of array manufacturing continuously. More detailed calibration data from laboratories or manufacturers’ manifests would be required, but might be difficult to obtain for arrays manufactured as far back as 1950.

Another key aspect to consider when using eMR-based data for epidemiological research is the potential enrichment of individuals with disease among individuals with eMR-based data^7,8^. This might be applicable to UK Biobank GP-clinical in the sense that individuals with suspected kidney impairment obtained more regularly creatinine measurements. While we found UK Biobank individuals with many eMR-records on creatinine to be less healthy than individuals with few records, we found little difference between participants that had eMR-records (i.e. were members of the GP-clinical) versus other participants. This might be due to the fact that serum creatinine is a typical routine biomarker, which is not only assessed upon indication for kidney impairment. The UK Biobank data on eGFR trajectories augmented with diagnostic codes from GP-clinical can be utilized for various aspects of analyses focusing on individuals with or without certain diagnostic codes.

In summary, we provide one approach to quantify bias in eMR-based creatinine measurements in UK Biobank that may be applicable also generally when gold standard measurements are available. We also provide a substantially extended longitudinal UK Biobank data resource on eGFR by integrating SC-based and bias-corrected eMR-based creatinine measurements that is usable for future research on eGFR decline. Our results may increase awareness of potential bias in past eMR-based biomarkers obtained before laboratory standards were available and when utilized assays were unknown. However, our results also underscore the opportunities from eMR-based data to enhance longitudinal information on kidney function, which is important for future studies on mechanisms of kidney function decline in the population.

## Electronic supplementary material

Below is the link to the electronic supplementary material.

Supplementary Material 1

## Data Availability

The datasets generated and/or analysed during the current study are available in the UK Biobank portal as return dataset (project number 20272, https://ams.ukbiobank.ac.uk/ams/).

## Abbreviations

eGFR: Estimated Glomerular Filtration Rate
eMR: Electronic Medical Record
SC: Study center
CKD-Epi: Chronic Kidney Disease Epidemiology Collaboration
GP: General Practitioner
crea_SC_: Serum creatinine obtained from study center
crea_eMR_: Serum creatinine obtained from electronic Medical Record
HbA1c: Hemoglobin A1c
CI: Confidence Interval
ESKD: End-stage Kidney Disease
AKI: Acute Kidney Injury
NIST: National Institute Standardized Technology
