# Supplementary material for: Bias-corrected serum creatinine from UK Biobank electronic medical records generates an important data resource for kidney function trajectories
Source: Sci Rep. 2025 Jan 28;15:3540. doi: 10.1038/s41598-025-85391-7 (PMC11775100; doi:10.1038/s41598-025-85391-7)
Supplement: Supplementary file 1 — Supplementary Material 1 [file 41598_2025_85391_MOESM1_ESM.docx]

# SUPPLEMENTARY ONLINE MATERIAL

# Bias-corrected serum creatinine from UK Biobank electronic medical records generates an important data resource for kidney function trajectories

**Supplementary Note 1** Quality control of serum creatinine measurements from eMR (crea_eMR_)

**Supplementary Note 2** Extraction of kidney-relevant events from GP-eMR

**Supplementary Table 1** Read terms for kidney function related diagnostic codes.

**Supplementary Table 2** Participant descriptives of GP-clinical members versus non-members.

**Supplementary Figure 1** Quality control of eMR-based serum creatinine measurements.

**Supplementary Figure 2** Difference between ln-transformed Study Center (SC) based creatinine and ln-transformed eMR-based creatinine values by calendar year of exam.

**Supplementary Figure 3** Comparison of eGFR derived from Study Center based creatinine (eGFR_SC_) with eGFR from eMR-based creatinine without bias-correction (eGFR_eMR_)

**Supplementary Figure 4** Comparison of eGFR derived from Study Center based creatinine (eGFR_SC_) with eGFR from eMR-based creatinine with bias-correction (eGFR_eMR_)

**Supplementary References**

# Supplementary Note 1: Quality control of serum creatinine measurements from eMR (crea_eMR_).

Read codes, specifically Clinical Terms Version 2 (Read v2) and Clinical Terms Version 3 (CTV3), are standardized vocabularies to codify clinical terms. In the UK healthcare system, individuals typically consult a GP, who refers patients to specialized care or requests biomarker measurements when indicated. Serum creatinine measurements are then conducted in contracted laboratories. The specific assays and protocols used for these measurements are unknown and presumed to vary between laboratories and time points.

To extract crea_eMR_ we used read codes (*1*). We quality-controlled the crea_eMR_ values for technical artefacts: (i) we excluded zero values. (ii) For creatinine values obtained on the same day (“duplicates”), we used the mean of natural logarithm of the two measures when the two measurements were within a 10% tolerance, excluded a value that was 88.4 times higher than the other (i.e. same value coded both in md/dL and μmol/L), or excluded both otherwise. (iii) We excluded values below the level of detection (LOD, 0.88 µmol/L, 0.01 mg/dL (*2*) or above the highest ever recorded value of 6524 µmol/L (73.8 mg/dL) (*3*). (iv) We set values below the level of quantification (LoQ, 4.42 µmol/L, 0.05 mg/dL (*2*) to the LoQ. This yielded our quality-controlled crea_eMR_ values (in μmol/L) and the date of recorded measurement. We assumed that this date was equivalent or close to the data of blood draw (i.e. “date-of-exam”, **Supplementary** **Figure 1**).

# Supplementary Note 2: Extraction of kidney-relevant events from GP-eMR.

To extract GP-eMR data on kidney-relevant events, we searched in the Coding System Lookups and Mappings Dictionary (Version 3, May 2021) for kidney-relevant events terms and identified the corresponding Read v2 and CTV3 read codes (**Supplementary Table 1**). Then, we extracted the GP-clinical information for these read codes. We merged an individual’s events data into the creatinine data according to the date-of-exam (i.e. date-of-exam for recorded event, date-of-exam for creatinine assessment).

For the GP-clinical released Sept 2019, the extraction date of eMR on behalf of the UK Biobank was between August 2016 and September 2017, depending on country (Scotland, Wales or England) and GP Computer System Supplier (EMIS, Vision or TPP). Thus, there is no serum creatinine value or kidney-relevant event after September 2017 in the presented data.

# Supplementary Table 1: Read terms for kidney function related diagnostic codes.

We extracted kidney function related information from UK Biobank “GP clinical” using these read codes. Read codes for raw creatinine and diabetes were obtained from Denaxas et al. (*1*), while all others were derived from the dictionary "Coding system lookups and mappings - Version 3, May 2021" (Read v2 or CTV3).

| **Diagnostic code** | **Read code v2 or CTV3 code** |
| --- | --- |
| Creatinine | "44J3.","44J30","44J31","44J32","44J33","44J3z","44JD.","44JC.","44J32","44J3z","44J33","44J31","44J30","XE2q5","XaERc","XaERX" |
| Diabetes | "1252.,"1434.","C10..,"C100.","C1000","C1000","C1000","C1001","C1001","C1001","C1001","C1001","C100z","C101.","C101.","C101.","C1010","C1010","C1011","C1011","C101y","C101z","C102.","C102.","C1020","C1021","C102z","C103.","C1030","C1030","C1031","C1031","C1031","C103y","C103z","C104.","C104.","C104.","C1040","C1041","C104y","C104z","C105.","C1050","C1051","C105y","C105z","C106.","C106.","C106.","C106.","C106.","C1060","C1061","C106y","C106z","C107.","C107.","C107.","C107.","C1070","C1071","C1072","C1073","C1074","C107y","C107z","C1080","C1080","C1080","C1081","C1081","C1081","C1082","C1082","C1082","C1083","C1083","C1083","C1085","C1085","C1085","C1086","C1086","C1086","C1087","C1087","C1087","C1087","C1088","C1088","C1088","C1089","C1089","C1089","C108y","C108z","C1090","C1090","C1090","C1091","C1091","C1091","C1092","C1092","C1092","C1093","C1093","C1093","C1094","C1094","C1094","C1095","C1095","C1095","C1096","C1096","C1096","C1096","C1097","C1097","C1097","C10A0","C10A1","C10A2","C10A3","C10A4","C10A5","C10A6","C10A7","C10B0","C10y.","C10y0","C10y1","C10yy","C10yz","C10z.","C10z0","C10z1","C10zy","C10zz","C11y0" |
| AKI | “XE2QM”,"X30Is","XaZ6J","XaZPp","XaZPs","XaZPt","XaZPu","XaPwv","XaZSx","X30Ir","XaZUY","XaZUZ","XaZUa","XaZYv","XaZZ0","XaZZ2","XaZe5","Xaa8O","Xaa8P","Xaa8Q","Y31fg","Yavic","Y31fh","Y31fn","Y31fo","Y31fr","Y31gQ","Y31gN","Y31gO","Y31fu","YavK2","YavVI","YavVL","YavVM","YavVN","Yasal","YavX4","YavYI","YavYJ","YavYK","YavYL","YavbY","Yavbb","Yavbd","Yavgj","Yaw12","Yaw13","Yaw14","Y31fj","Y31fi","K04..","K040.","K041.","K042.","K043.","K0430","K0431","K0432","K0433","K0434","K044.","K045.","K046.","K0460","K0461","K047.","K048.","K049.","K04A.","K04B.","K04C.","K04D.","K04E.","K04y.","K04z." |
| ESKD | “K05..”,"K050.","K055.","X30J0","Y31gA","XaLHK","YaoLB" |
| Nephrectomy | “7B01.”,"7B01.","7B010","7B010","7B010","7B011","7B012","7B013","7B013","7B014","7B015","7B015","7B016","7B017","7B018","7B019","7B01y","7B01z","7B02.","7B02.","7B020","7B021","7B022","7B023","7B02y","7B02z","7B01.","7B01.","7B010","7B010","7B010","7B011","7B012","7B013","7B013","7B014","XE0Fv","Xa2h5","7B016","7B017","7B018","Xa2h5","7B01y","7B01z","7B02.","7B02.","7B020","7B021","7B022","7B023","7B02y","7B02z","Y30ez","Y30f0","Y30f3","Y30f3","Y30f4","Y30f6","Y30fA","Y30fM","Y30fN","Y30fB","Y30fD","Y30fE","Y30fF","Y30f7","Y30f8","Y30fE","Y30f2","Y30f1","Y30fH","Y30fI","Y30fL","Y30fO","Y30fQ","Y30fS","Y30fK","Y30fJ" |
| Dialysis | “.14V2”,"14V2.","Ya07N","XE0Jg","Xa8S7","Xa8S7","Ya0Wc","7L1A.","7L1A0","7L1A1","7L1A2","7L1A3","7L1A4","7L1A5","7L1A6","7L1A7","XE0Jf","Y75Sk","YMIKl","Ya15a","Y03dD","Y03dH","7L1A1","7L1A2","X01AL","XaM2A","X40c3","XaMMt","X01AV","Y42A4","YaV79","Y03dQ","YapAD","Y42A6","YapYh","Y03dh","Y03c7","8882.","Y74u7","TB11." |
| Kidney Transplant | “7B00.”,"7B000","7B001","7B001","7B002","7B002","7B002","7B002","7B002","7B003","7B004","7B005","7B006","7B00y","7B00z","XaM1o","XaM1p","XaMKM","X30D2","Y30ef","Y30ew","Y30eq","Y30er","Y30el","Y30ep","Y30el","Y30ep","Y30el","Yap9l","Yap9m","YapW3","Y30ev","Y30ek","Y30ej","TB001","Y74uZ","ZV420","Ya0O1" |
| Pregnancy | “615C.”,"Y79tL","Y79tM","62...","6217.","6218.","6219.","621A.","621B.","621C.","621D.","621Z.","Y7EF5","Y79Qh","Y79Qj","X74V6","Y79Qi","621..","Y7EF7","Y7EF6","6211.","Y7EFF","6212.","Y7EFE","6213.","Y7EFD","6214.","Y7EFC","6215.","Y7EFB","6216.","Y7EFA","Y7EEZ","Y7EEY","Y7EEN","Y7EEX","Y7EEU","X76Qo","Y7EEV","X40Ah","Y40j9","Y7EF9" |

We obtained the read codes for creatinine and Diabetes from Denaxas et al. AKI=Acute Kidney Injury, ESKD=End-stage Kidney Disease.

# Supplementary Table 2: Participant descriptives of General Practitioner (GP)-clinical members versus non-members.

We show participant characteristics using the information from the study center (SC) visit at baseline and descriptive statistics of the combined SC- and (bias-corrected) eMR-based eGFR (*4*). This is shown for non-members and members of “GP-clinical” and, for GP-clinical members, separately for individuals with 1-8 and ≥9 eMR-based eGFR assessments (i.e. comparable to individuals with 2-9 versus ≥10 eGFR assessments for eMR- and SC-based eGFR combined, since most individuals have one SC-based eGFR). ‘n’ denotes the number of individuals and ‘m’ the number of eGFR assessments. Presented are mean and standard deviations, if not stated otherwise.

|  | **Non-members GP-clinical**  **(n=255,511, m=265,689)** | **Members**  **GP-clinical**  **1-8 eMR-eGFR**  **(n=120,952, m=521,535)** |  | **Members**  **GP-clinical**  **≥9 eMR-eGFR**  **(n=78,444, m=1,314,950)** | |  |
| --- | --- | --- | --- | --- | --- | --- |
| **Characteristics from SC-baseline*** | | | | |  | |
| Age - years | 57.0 ± 8.2 | 55.8 ± 8.0 |  | 60.5 ± 7.0 | |  |
| Sex – female (%) | 137,993 (54.0) | 69,280 (57.3) |  | 39,207 (50.0) | |  |
| Ancestry - European (%) | 242,813 (95.0) | 103,725 (85.8) |  | 67,559 (86.1) | |  |
| Smoking status **– current or ever (%) | 113,508 (44.5) | 51,605 (42.7) |  | 38,938 (49.7) | |  |
| BMI **– kg/m² | 27.3 ± 4.7 | 26.8 ± 4.4 |  | 29.0 ± 5.2 | |  |
| Diabetes – yes (%) | 10,249 (4.2) | 832 (0.7) |  | 8,398 (11.4) | |  |
| eGFR - mL/min/1.73m² | 94.3 ± 13.1 | 95.9 ± 11.9 |  | 90.8 ± 14.4 | |  |
| Chronic Kidney Disease**** – yes (%) | 4,176 (1.6) | 695 (0.7) |  | 2,387 (3.4) | |  |
| **Combined SC- and eMR-based assessments** | | | | |  | |
| Age-first-exam^‡^ - years | 56.8 ± 8.2 | 53.9 ± 8.4 |  | 55.6 ± 7.4 | |  |
| Age-last-exam^‡^ - years | 57.0 ± 8.2 | 60.8 ± 8.5 |  | 67.3 ± 7.0 | |  |
| time between 1^st^ and last eGFR assessment - median (max) - years | 0.0 (6.1) | 6.8 (40.0) |  | 11.5 (60.2) | |  |
| Calendar year of exam^‡^ - min to max | 2007 – 2013 | 1970 – 2017 |  | 1950 – 2017 | |  |
| #eGFR assessments per person – median [IQR] | 1.0 [1.0 - 1.0] | 4.0 [3.0 - 6.0] |  | 14.0 [11.0 – 19.0] | |  |
| eGFR-first-exam - mL/min/1.73m² | 94.5 ± 13.1 | 96.9 ± 12.1 |  | 94.3 ± 13.2 | |  |
| eGFR-last-exam - mL/min/1.73m² | 94.3 ± 13.2 | 90.2 ± 12.9 |  | 81.9 ± 16.3 | |  |
| Chronic Kidney Disease*** – yes (%) | 4,126 (1.6) | 3,871 (3.2) |  | 15,038 (19.1) | |  |

BMI=Body Mass Index, eGFR=estimated Glomerular Filtration Rate.
* obtained from SC-baseline (data fields 31, 34, 52, 53, 2443, 20003, 20116, 21001, 30700, and 30750).

** Among individuals where this variable is available (n=454,361 for smoking status 0=never smoking, 1=current or previous smoking, n=452,721 for BMI).

*** Individuals having eGFR <60 mL/min/1.73m² for at least one timepoint

^‡^ “Exam” is the examination in SC or the examination by the GP, assuming that the date of the eMR-record is the same date as the GP-exam.

# Supplementary Figure 1: Quality control of eMR-based serum creatinine measurements.

We extracted all eMR-based serum creatinine measurements recorded in GP-clinical using an approach and read codes provided by Denaxas et al. (*1*). Shown are the number of individuals and measurements (‘n’ and ‘m’ in boxes) at each step of the quality control. Our quality control process involved (i) excluding zero values of creatinine and eliminating duplicates, (ii) excluding same-day measurements, (iii) excluding values below the Limit of Detection (LoD), (iv) setting values below the Limit of Quantification (LoQ) to the LoQ and (v) excluding implausible high values. Example eGFR values for thresholds of creatinine values were given for a 50-year-old man (*4*).


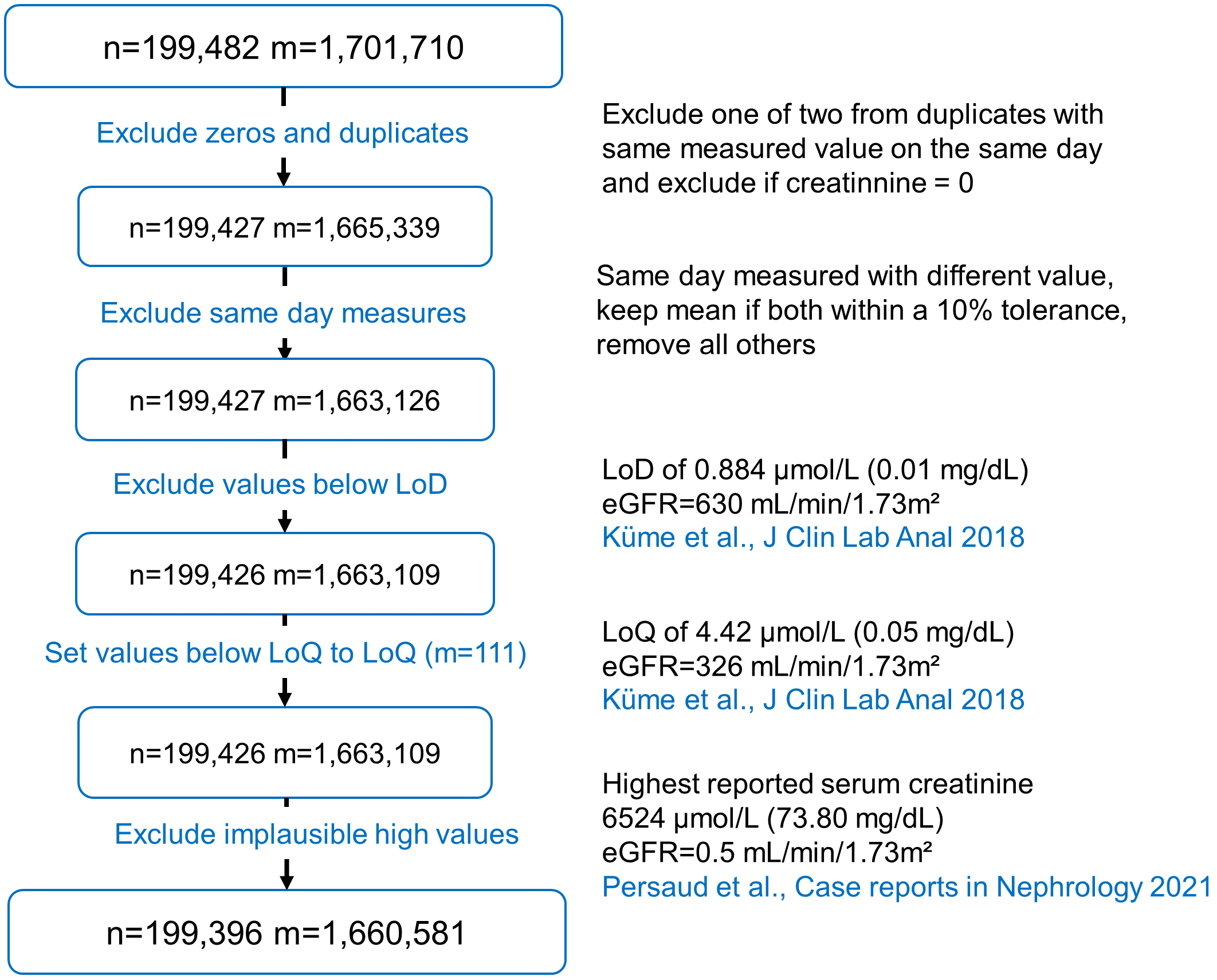


# Supplementary Figure 2: Difference between ln-transformed Study Center (SC)-based creatinine and ln-transformed electronic Medical Records (eMR)-based creatinine values by calendar year of exam.

We evaluated whether eMR-based creatinine (crea_eMR_) values were comparable to SC-based creatinine (crea_SC_) values in individuals with crea_eMR_ and crea_SC_ obtained in the same calendar (sample size ‘n’ given above each panel, n overall=70,231). We considered crea_SC_ values gold standard. We show the individuals’ difference between ln(crea_SC_) and ln(crea_eMR_) versus ln(crea_SC_). Sex is indicated by color-coding (blue for men, pink for women). Horizontal grey line represents the null-line. Red dashed lines represent mean difference between ln(crea_SC_) and ln(crea_eMR_). Black dashed lines represent corresponding 95% confidence intervals, indicating range of values on the y-axis. Stated are, in each panel, s^year^ and “bias” derived as exp(s^year^) (see also **Figure 2B**).


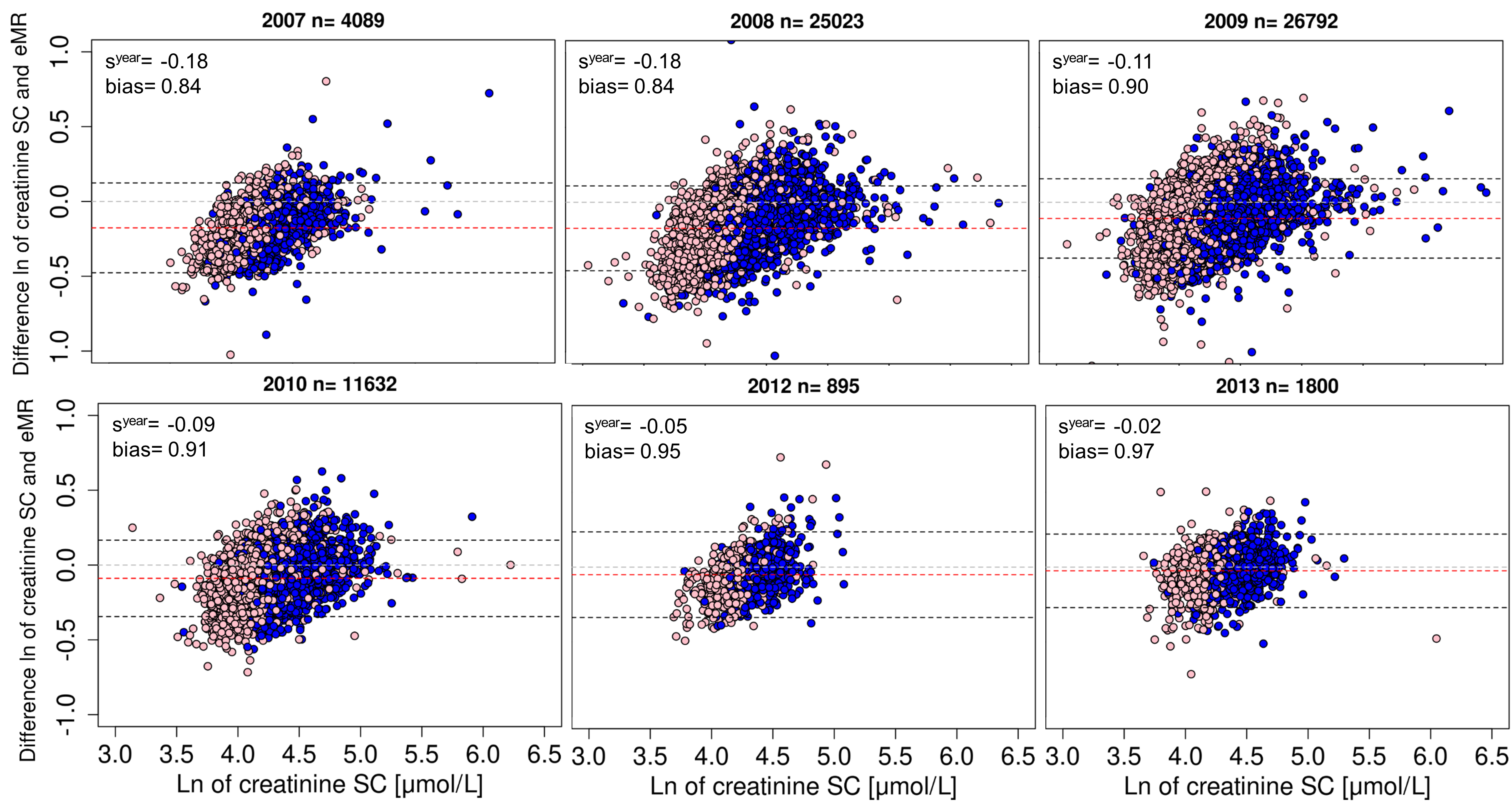


# Supplementary Figure 3: Comparison of eGFR derived from Study Center based creatinine (eGFR_SC_) with eGFR derived from electronic Medical Records (eMR)-based creatinine (eGFR_eMR_) without bias-correction.

We show eGFR_SC_ versus eGFR_eMR_ (quality-controlled, not bias-corrected) among 70,231 individuals with both eGFR assessment from the same calendar year (using the eGFR_eMR_ closest to the eGFR_SC_) with sex indicated by color (blue for men, pink for women). We estimated eGFR via the CKD-EPI 2021 formula (*4*). Red dotted line indicates identity.


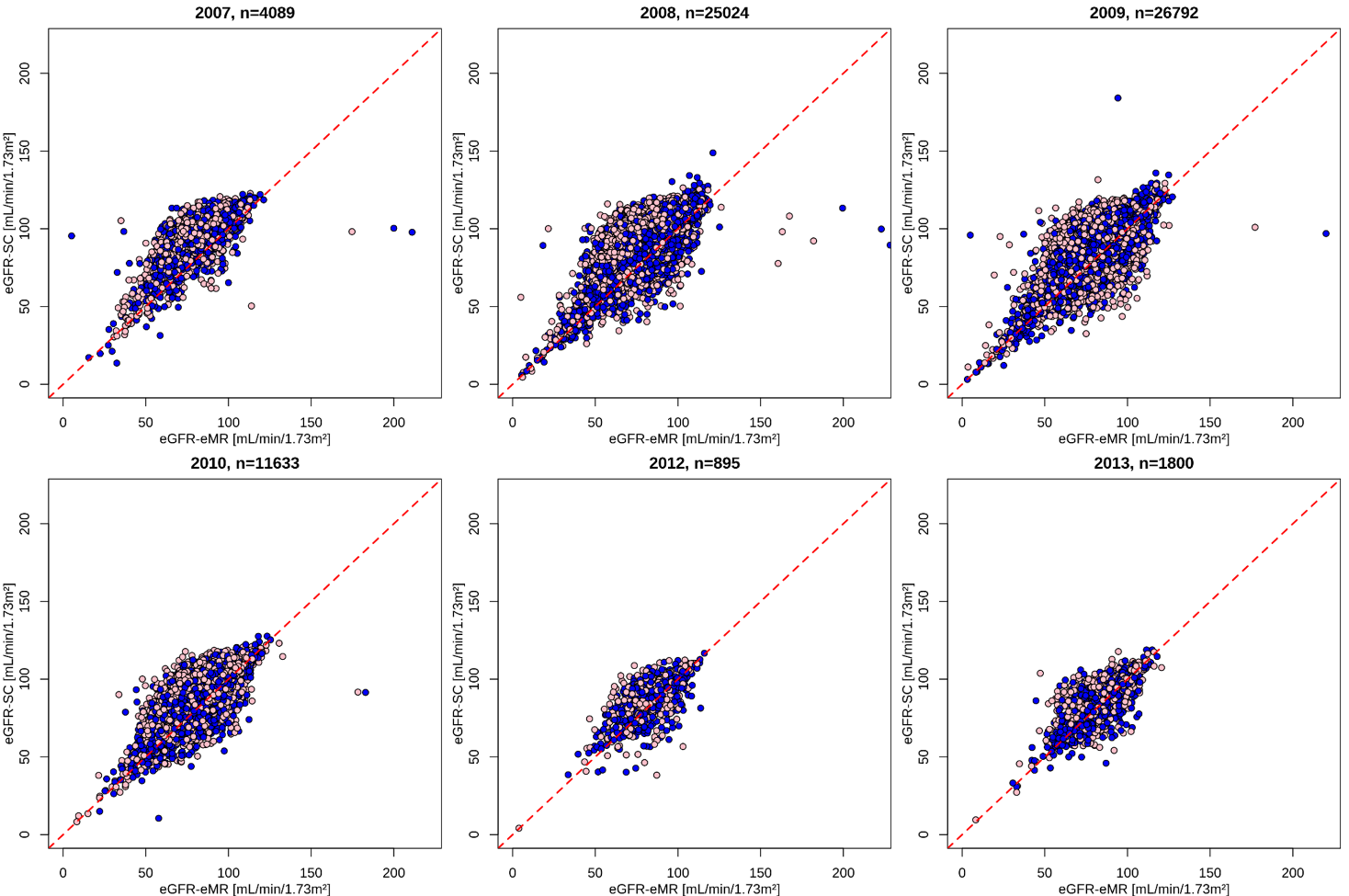


# Supplementary Figure 4: Comparison of eGFR derived from Study Center (SC) based creatinine (eGFR_SC_) with eGFR derived from electronic Medical Records (eMR)-based creatinine (eGFR_eMR_) with bias-correction.

We show eGFR_SC_ versus eGFR_eMR_ (quality-controlled, bias-corrected) among 70,231 individuals with both eGFR assessment from the same calendar year (using the eGFR_eMR_ closest to the eGFR_SC_) with sex indicated by color (blue for men, pink for women). We estimated eGFR via the CKD-EPI 2021 formula (*4*). Red dotted line indicates identity.


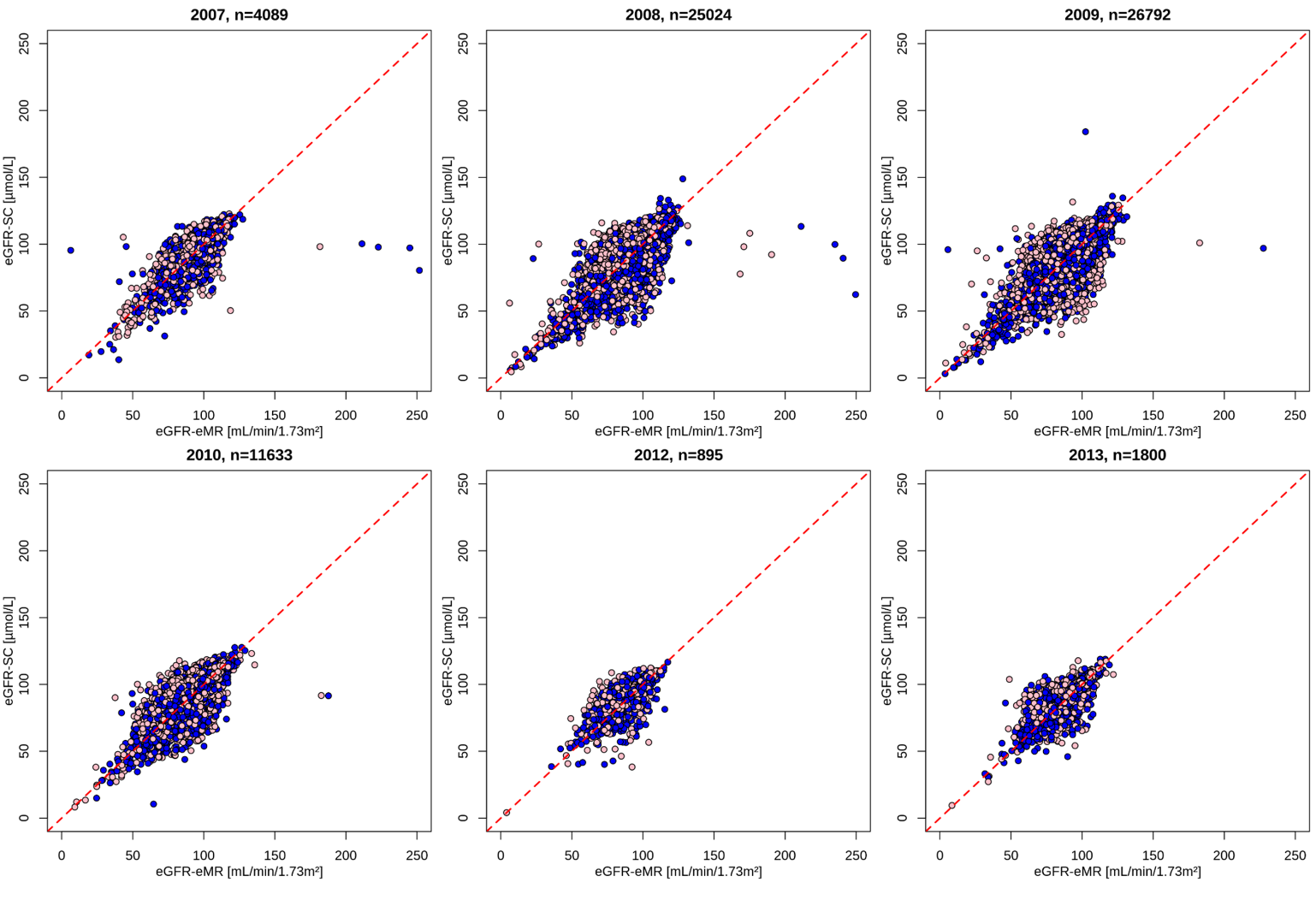


Supplementary ReferencesReferences

1. Denaxas S, Shah AD, Mateen BA, Kuan V, Quint JK, Fitzpatrick N, et al. A semi-supervised approach for rapidly creating clinical biomarker phenotypes in the UK Biobank using different primary care EHR and clinical terminology systems. JAMIA Open 2020;3:545–56.

2. Küme T, Sağlam B, Ergon C, Sisman AR. Evaluation and comparison of Abbott Jaffe and enzymatic creatinine methods: Could the old method meet the new requirements? J Clin Lab Anal 2018;32.

3. Persaud C, Sandesara U, Hoang V, Tate J, Latack W, Dado D. Highest Recorded Serum Creatinine. Case Reports in Nephrology 2021;2021:1–3.

4. Inker LA, Eneanya ND, Coresh J, Tighiouart H, Wang D, Sang Y, et al. New Creatinine- and Cystatin C-Based Equations to Estimate GFR without Race. N Engl J Med 2021;385:1737–49.
